# PARTICIPATION AND SOCIAL CONNECTIONS IN A RECREATIONAL SENIOR SOFTBALL LEAGUE

**DOI:** 10.1093/geroni/igz038.1968

**Published:** 2019-11-08

**Authors:** Dawn S Tarabochia

**Affiliations:** Montana State University, Bozeman, Montana, United States

## Abstract

Senior only communities have long been an option for adults over a certain age. A variety of activities and clubs are often available to residents of these communities. The purpose of this research project was to understand the lived experience of recreational softball players regarding players decision to play senior softball and to determine what social opportunities were associated with recreational senior softball leagues. A phenomenological research study was constructed to seek further inquiry into two research questions associated with this project. Participants were members of a senior living community and members of a recreational senior softball league. Convenience and snowball sampling techniques were utilized, and 25 interviews were conducted. The interview transcripts were analyzed for phenomenological themes by the research team. The researchers used Van Manen’s (1990) hermeneutical approach to analyze data. Trustworthiness was established by the use of a peer reviewer to assess the themes for accuracy. Themes associated with the first research question indicate that interviewees participated in softball for a variety of reasons, including having played softball as a younger adult, wanting to maintain a level of physical fitness, and for the social connections that participation in a softball league provided. Themes associated with the second research question found that participants enjoyed many social benefits from playing softball, including informal and formal social opportunities. In conclusion, the willingness and opportunity to play senior softball provided older adults the ability to maintain a certain level of physical activity and to engage in meaningful social interactions.

